# Altered cytokine pattern and inflammatory pathways in monogenic and complex autoinflammatory diseases

**DOI:** 10.1186/1546-0096-13-S1-O48

**Published:** 2015-09-28

**Authors:** P Galozzi, O Negm, P Sfriso, P Tighe, I Todd, L Punzi

**Affiliations:** 1 University of Padova, Department of Medicine DIMED, Padova, Italy; 2 University of Nottingham, Department of Immunology, Nottingham, UK

## Introduction

Autoinflammatory disorders (AIDs) are a group of innate immunity-related diseases, characterized by seemingly unprovoked inflammatory episodes mainly involving skin, eyes and joints. They can be categorized into hereditary monogenic and multifactorial complex disorders. The inflammatory mechanisms underlying both monogenic and complex AIDs are not completely understood.

## Objectives

In order to clarify them, we started to evaluate the *ex vivo* cytokine profile and the activation of the principal inflammatory pathways in Familial Mediterranean Fever (FMF), TNF-receptor associated periodic syndrome (TRAPS), Blau syndrome (BS) and Adult Onset Still's Disease (AOSD) patients during attack-free periods, and compare the results with those from healthy controls.

## Patients and methods

The study included 7 FMF, 12 TRAPS, 2 BS, and 8 AOSD patients and 27 healthy controls. Cytokine levels were evaluated by Antibody microarray in serum, whereas pathway activation through Reverse phase protein array (RPPA) in peripheral blood mononuclear cells (PBMCs).

## Results

Interleukin (IL)-17, IL-22, and IL-23 were all significantly raised in our cohort of AIDs compared to controls, whereas IL-1β, IL-6, IL-8, and TNF-α levels were differently heighten among the diseases. Thus, the cytokine pattern may help to distinguish the AIDs in term of number of enhanced cytokines, as follow BS<TRAPS<FMF<AOSD. Moreover, the upregulation of Th17-related cytokines may suggest an important role for Th17 or Th17-like cells in AIDs, that are directly involved in inflammatory processes.

All diseases patients presented a general constitutive activation of inflammatory pathways compared to the control group. In particular, NF-κB, MAPK, PI3K/AKT, JAK/STAT and NLRP1 inflammasome pathways were all upregulated in AOSD, whereas TRAPS, FMF and BS show different specific activation. So, the variance among the results suggested a more complicated relationship between individual patients and pathways compounds, associated with their peculiar clinic and genetic conditions.

## Conclusion

Our results suggested an ongoing subclinical inflammation related with the abnormal and constitutive signalling pathways and defined an elevated inflammatory cytokine signature.

Thus, if confirmed, the evaluation of the number of raised cytokines could be a new way to stratify autoinflammatory diseases patients. Furthermore, critical in AIDs may be the modulation of the Th17 cytokine network pathway.

